# Clinical Characteristics, Etiology and Antimicrobial Susceptibility among Overweight and Obese Individuals with Diarrhea: Observed at a Large Diarrheal Disease Hospital, Bangladesh

**DOI:** 10.1371/journal.pone.0070402

**Published:** 2013-08-01

**Authors:** Sumon Kumar Das, Mohammod Jobayer Chisti, Sayeeda Huq, Mohammad Abdul Malek, Lana Vanderlee, Guddu Kaur, Mohammed Abdus Salam, Tahmeed Ahmed, Abu Syed Golam Faruque, Abdullah Al Mamun

**Affiliations:** 1 International Centre for Diarrheal Disease Research, Bangladesh, Dhaka, Bangladesh; 2 School of Public Health and Health Systems, University of Waterloo, Waterloo, Canada; 3 The University of Sydney, Sydney, Australia; 4 School of Population Health, University of Queensland, Brisbane, Australia; University of Sao Paulo, Brazil

## Abstract

**Background:**

The present study aimed to determine the clinical characteristics and etiology of overweight and obese (OO) individuals with diarrhea attending an urban Dhaka Hospital, **International Centre for Diarrheal Disease Research** (icddr,b), Bangladesh.

**Methods:**

Total of 508 under-5 children, 96 individuals of 5–19 years and 1331 of >19 years were identified as OO from the Diarrheal Disease Surveillance System (DDSS) between 1993–2011. Two comparison groups such as well-nourished and malnourished individuals from respective age stratums were selected.

**Results:**

Isolation rate of rotavirus was higher among OO under-5 children compared to malnourished group (46% vs. 28%). Rotavirus infection among OO individuals aged 5–19 years (9% vs. 3%) (9% vs. 3%) and >19 years (6% vs. 4%) (6% vs. 3%) was higher compared to well-nourished and malnourished children. Conversely, *Vibrio cholerae* was lower among all OO age groups compared to well-nourished and malnourished ones. *Shigella* (4% vs. 6%) (4% vs. 8%), and *Campylobacter* (3% vs. 5%) (3% vs. 5%) were lower only among OO in >19 years individuals compared to their counterparts of the same age stratum. *Salmonella* was similarly isolated in all age strata and nutritional groups. In multinomial logistic regression among under-5 children, significant association was observed only with use of antimicrobials at home [OR-1.97] and duration of hospital stay [OR-0.68]. For individuals aged 5–19 years, use of antimicrobials at home (OR-1.83), some or severe dehydration (OR-3.12), having received intravenous saline (OR-0.46) and rotavirus diarrhea (OR-2.96) were found to be associated with OO respectively. Moreover, significant associations were also found for duration of diarrhea before coming to hospital (>24 hours) (OR-1.24), *Shigella* (OR-0.46), and *Campylobacter* (OR-0.58) among >19 years OO individuals along with other associated co-variates in 5–19 years group (all p<0.05).

**Conclusion and significance:**

Higher proportion of OO were infected with rotavirus and a greater proportion of them used antimicrobials before coming to the hospital.

## Introduction

Diarrhea remains a major public health problem globally [1], [2]. Diarrheal diseases are a leading cause of morbidity and deaths in developing countries [3], [4]. Obesity is a public health concern that is also associated with substantial morbidity [5], [6], [7] and negatively impacts the quality of life [8], [9]. The global prevalence of overweight and obesity has increased steadily over the last three decades [10], [11], [12], [13]. Although obesity is generally believed to be a problem amongst people in higher or middle class living in urban areas, the burden has started shifting towards the poor and in rural population [14].

Most recent studies have focused on the association between overweight and obesity with chronic diseases [12], [13], [15], [16], [17], [18]. However, diarrhea is often experienced among overweight and obese individuals irrespective of age, contributing to the double burden of disease seen in many low-income countries. There is lack of information about the prevalence and characteristics of diarrhea among the overweight and obese population in many developing countries including Bangladesh. Additionally, research on diarrheal disease mostly concentrates on children with an emphasis on childhood malnutrition [19], [20]. Within this framework, the present study aimed to determine clinical characteristics as well as common etiology of diarrhea with their antimicrobial susceptibility among overweight and obese individuals. The study also compared the findings with non-overweight and non-obese individuals such as well-nourished and malnourished groups.

## Methods

### Study Site, Source of Data and the Hospital Surveillance System

The study population included residents of urban or peri-urban areas of Dhaka who presented with diarrhea to Dhaka Hospital of the International Centre for Diarrhea Disease Research, Bangladesh (icddr,b). Established in 1962, this hospital currently provides free care and treatment to approximately 140,000 patients each year. A Diarrheal Disease Surveillance System (DDSS) has been used for the extraction of our data from 1993–2011. The detailed information of sampling process of DDSS has already been described in previously published article [21], [22] which is same for this manuscript.

### Definition

Most of the studies of recent past related to diarrheal illnesses focused on young under-5 children because they are at highest risk for morbidity and mortality. To determine the clinical characteristics and etiologic diversity, overweight and obesity was defined following the WHO guidelines [23], [24], and stratified patients under three age stratums: (i) children under 5 years of age, (ii) people aged 5–19 years, and (iii) people older than 19 years. Overweight and obese were defined for each age category (weight-for-age z-score for under-5 children [≥ +2.00 SD], BMI for age z-score for individuals aged 5–19 years [≥+2.00 SD]; and BMI for those older than 19 years [≥25 kg/m^2^]). Well-nourished and malnourished were defined as followed: less than 5 years [weight-for-age z-score (≥−2.00 to <+1.00 SD; <−2.00 SD)], 5–19 years [BMI-for-age z-score (≥−2.00 to +1.00 SD; <−2.00 SD)] and above 19 years [BMI (18.5 to <25 kg/m^2^; <18.5 kg/m^2^)], respectively.

### Sampling Frame

**Figure 1:**
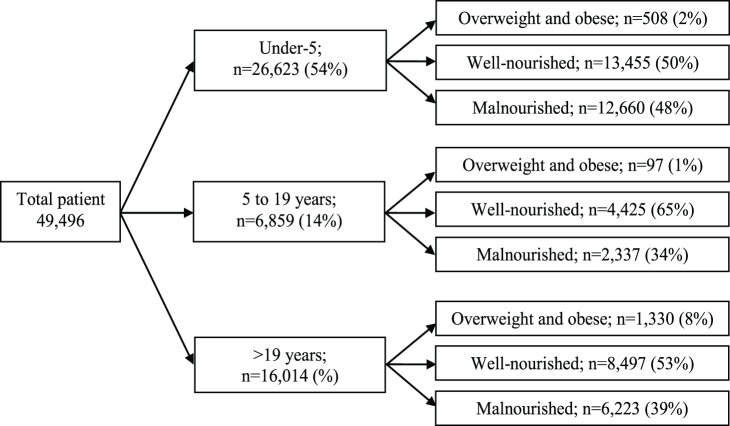
Sample framing.

In total, 49,496 patients were enrolled in the DDSS during the study period (1993–2011). Out of them, 26,623 were children less than 5 years old, 6,859 were individuals aged 5–19 years and 16,014 were individuals older than 19 years. Among these three populations, overweight and obesity together represented 2% (n = 508), 1% (n = 97) and 8% (n = 1330) respectively. On the other hand, among the well-nourished groups, the distributions were 13,455 (50%); 4,425 (65%); 8,497 (53%) and for malnourished those were 12,660 (48%); 2,337 (34%); 6,223 (39%) for each age group, respectively (Figure 1).

### Lab Methodology

Each stool specimen was aliquoted into three serial containers and submitted to the respective laboratories of icddr,b for culture and molecular analysis. Enterotoxigenic *Escherichia coli* (ETEC) [25] was detected using singleplex Polymerase Chain Reaction (PCR). *Vibrio cholerae* [26], *Shigella* spp. [27], [28], *Salmonella* [27], [28], and *Campylobacter* [27], [28] were isolated and characterized using standard bacteriological culture method in the Clinical Microbiology Laboratory. Rotavirus [29] was identified using ELISA method.

Antimicrobial susceptibility was performed using disc diffusion method and susceptibility pattern was defined following Clinical and Laboratory Standards Institute (CLSI) guideline. Testing of *Shigella* isolates was performed against ampicillin, cotrimoxazole, nalidixic acid, and ciprofloxacin. Susceptibility for *Vibrio cholerae* was tested for azithromycin (15 mg), ciprofloxacin (5 mg), erythromycin (15 mg), tetracycline (30 mg) and cotrimoxazole (trimethoprim-sulfamethoxazole) (25 mg) [30].

### Data Analysis

We analysed data using Statistical Package for Social Sciences (SPSS) Windows (Version 15.2; Chicago, IL) and Epi Info (Version 6.0, USD, Stone Mountain, GA). We compared differences in the proportion using Chi-square test and a probability of <0.05 was considered as statistically significant. Strength of association was determined by estimating odds ratio (OR) and 95% confidence intervals (CI). Estimated number of patients was calculated by multiplying the number of patients enrolled by 50 (2% sample). Then proportions were calculated. Finally, multinomial logistic regression analysis was performed considering well-nourished as reference group. In the analysis, clinical manifestation and etiology of diarrhea which were identified to be associated in univariate analyses were considered in the multinomial models to determine the independent association with the outcome variable.

### Ethical Statement

The Diarrheal Disease Surveillance System (DDSS) of icddr,b is a routine ongoing activity of the Dhaka Hospital, which has been approved by the Research Review Committee (RRC) and Ethical Review Committee (ERC) of icddr,b. At the time of enrollment, verbal consent was taken from the caregivers or guardians on behalf of the patients. The information to be stored in the hospital database and used for conducting researches. Although the DDSS of icddr,b is a scheduled activity on the hospital patients, and used to be performed after taking verbal consent from the parents or guardians of the patients following the hospital policy. This verbal consent was documented by keeping a check mark in the questionnaire which was again shown to the patient or the parents. Parents or guardians were assured about the non-disclosure of information collected from them, and were also informed about the use of data for analysis and using the results for improving patient care activities as well as publication without disclosing the name or identity of their children. ERC was satisfied with the voluntary participation, maintenance of the rights of the participants and confidential handling of personal information by the hospital physicians and has approved this consent procedure.

## Results

**Figure 2:**
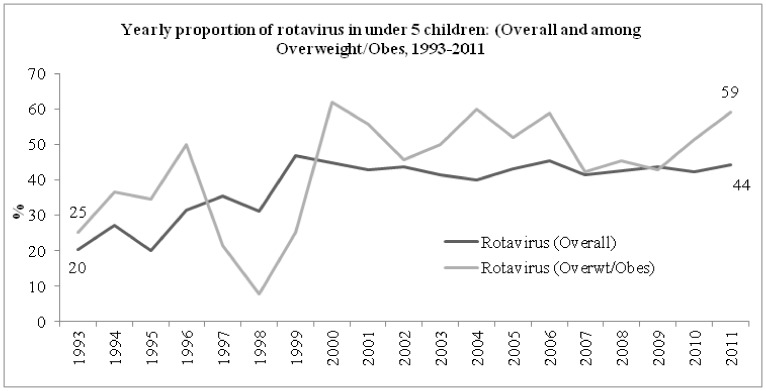
Yearly distribution of rotavirus (overall and overweight and obese) among under-5 *children*.

Among all children under 5 years old enrolled in the DDSS, the proportion of overweight and obesity increased from 0.64% in 1993 to 5.15% in 2011 (chi-square for trend <0.001). Similarly, among those 5–19 years enrolled, the proportion increased from 0.80% to 6.70% (chi-square for trend <0.001), and 3.66% to 16.94% (chi-square for trend <0.001) among those in the oldest age category (>19 years) during the same period. From 1993 to 2011, the proportion of overall rotavirus increased from 20% to 44% among children under-5 years. Simultaneous increase of rotavirus among overweight and obese children was also noted from 25% to 59% with a drop in 1998 (Figure 2).

Overall isolation rate among overweight and obese individuals was *Vibrio cholerae* 18%, *Shigella* 4%, *Salmonella* 2%, *Campylobacter* 4%, and rotavirus 17%. For well-nourished, it was 14%, 6%, 2%, 6%, 24%, and for malnourished individuals it was 20%, 7%, 2%, 8%, 18% respectively.

Vomiting was less frequently observed among overweight and obese under-5 children compared to well-nourished as well as malnourished under-5 children (Table 1). A significantly higher proportion of overweight and obese under-5 children passed stool more than 10 times per day compared to other groups. Although the proportion of children attending the triage area with longer duration of diarrhea (more than 24 hours) was similar among all the groups, a smaller proportion of overweight and obese children remained in the hospital for long duration compared to the other two groups as observed in univariate and multivariate analysis (Table 1). The isolation rate of *Vibrio cholerae* was lower among overweight and obese children than well-nourished and malnourished children, and the isolation rate of rotavirus was higher; however, the rates of isolation of *Shigella, Salmonella,* and *Campylobacter* were not significantly different among overweight and obese, well-nourished and malnourished groups. The proportion of children who received antimicrobials before attending the hospital was higher among overweight and obese children compared to the counterparts in the univariate and multinomial logistic regressions (Table 1).

**Table 1:** Comparative and multinomial logistic regression analysis of characteristics and common etiology of diarrhea among well-nourished with overweight and obese, and malnourished children under 5 years.

Indicators	Overweightand obesen = 508 (%)	Well- nourishedn = 13,455 (%)	Malnourishedn = 12,660 (%)	Overweight and obese vs. well-nourished		Malnourished vs. well-nourished	
				Unadjusted OR (95% CI)p value	Adjusted OR (95% CI)p value	Unadjusted OR (95% CI)p value	Adjusted OR (95% CI)p value
Antimicrobial therapy before attending the hospital	260 (51)	4464 (33)	2649 (21)	2.11 (1.76, 2.53) <0.001	1.97 (1.55, 2.50) <0.001	0.53 (0.50, 0.56) <0.001	0.53 (0.49, 0.57) <0.001
Fever (≥37.8°C)	29 (6)	941 (7)	1208 (10)	0.81 (0.54, 1.19) 0.303	0.73 (0.47, 1.15) 0.178	1.40 (1.28, 1.53) <0.001	1.28 (1.14, 1.44) <0.001
Vomiting	361 (71)	10232 (76)	9971 (79)	0.77 (0.63, 0.95) 0.012	0.86 (0.68, 1.08) 0.208	1.17 (1.10, 1.24) <0.001	1.08 (0.99, 1.17) 0.059
Diarrhea duration of >1 day	393 (77)	10170 (76)	9771 (79)	1.10 (0.89, 1.37) 0.388	0.88 (0.68, 1.15) 0.359	1.09 (1.03, 1.16) 0.003	1.38 (1.27, 1.49) <0.001
Stool frequency >10/24 hours	228 (45)	5339 (40)	5206 (41)	1.24 (1.03, 1.48) 0.021	1.21 (0.97, 1.50) 0.085	1.06 (1.01, 1.12) 0.018	0.89 (0.83, 0.95) 0.891
Watery stool	477 (94)	12635 (94)	11727 (93)	1.00 (0.68, 1.47) 0.931	0.95 (0.62, 1.44) 0.792	0.82 (0.74, 0.90) <0.001	0.77 (0.67, 0.88) <0.001
Abdominal pain	119 (23)	2913 (22)	2662 (21)	1.11 (0.89, 1.37) 0.369	1.08 (0.84, 1.39) 0.533	0.96 (0.91, 1.02) 0.225	0.88 (0.81, 0.95) 0.003
Dehydration (some or severe)	104 (21)	3188 (24)	5184 (41)	0.83 (0.66, 1.04) 0.104	1.01 (0.75, 1.36) 0.942	2.23 (2.12, 2.36) <0.001	1.65 (1.52, 1.79) <0.001
IV rehydration	17 (3)	900 (7)	2360 (19)	0.48 (0.29, 0.80) 0.004	0.53 (0.26, 1.12) 0.092	3.20 (2.94, 3.47) <0.001	1.68 (1.48, 1.91) <0.001
Hospitalization >24 hrs.	125 (25)	4589 (34)	6569 (52)	0.63 (0.51, 0.78) <0.001	0.68 (0.53, 0.88) 0.004	2.08 (1.98, 2.19) <0.001	1.97 (1.84, 2.12) <0.001
Isolation of pathogens							
* V. cholerae*	17 (3)	948 (7)	1629 (13)	0.46 (0.27, 0.76) 0.001	0.67 (0.37, 1.22) 0.192	1.95 (1.79, 2.12) <0.001	0.99 (0.88, 1.13) 0.956
* Shigella*	25 (5)	696 (5)	878 (7)	0.95 (0.62, 1.45) 0.881	0.80 (0.49, 1.32) 0.383	1.46 (1.31, 1.62) <0.001	1.13 (0.98, 1.30) 0.093
Rotavirus	235 (46)	5730 (43)	3501 (28)	1.16 (0.97, 1.39) 0.110	1.08 (0.86, 1.35) 0.493	0.52 (0.49, 0.54) <0.001	0.57 (0.54, 0.62) <0.001
* Salmonella*	4 (<1)	154 (1)	241 (2)	0.69 (0.22, 1.92) 0.594	0.94 (0.34, 2.58) 0.904	1.68 (1.36, 2.07) <0.001	1.57 (1.21, 2.06) 0.001
* Compylobactar*	34 (7)	925 (7)	1136 (9)	0.97 (0.67, 1.40) 0.944	0.99 (0.66, 1.49) 0.958	1.34 (1.22, 1.46) <0.001	1.32 (1.18, 1.48) <0.001

N.B: Well-nourished considered as reference category.

Among the 5–19 years age stratum, use of antimicrobials before attending the hospital, frequency of passage of stool more than 10 times per day, intravenous saline administration for correction of dehydration, *Vibrio cholerae* and rotavirus were found to be significantly associated with obesity and overweight in univariate analysis. Fewer variables were significant in multinomial analysis, including some or severe dehydration, use of antimicrobials before attending the hospital, frequency of passage of stool more than 10 times per day and rotavirus were found to be associated with overweight and obesity (Table 2).

**Table 2:** Comparative and multinomial logistic regression analysis of characteristics and common etiology of diarrhea among well-nourished with overweight and obese, and malnourished individuals aged 5–19 years.

Indicators	Overweightand obesen = 97 (%)	Well- nourished n = 4,425 (%)	Malnourishedn = 2,337 (%)	Overweight and obese vs. well-nourished		Malnourished vs. well-nourished	
				Unadjusted OR (95% CI)p value	Adjusted OR (95% CI)p value	Unadjusted OR (95% CI)p value	Adjusted OR (95% CI)p value
Antimicrobial therapy before attending the hospital	26 (27)	642 (15)	268 (12)	2.16 (1.33, 3.48) <0.001	1.83 (1.05, 3.16) 0.031	0.76 (0.65, 0.89) 0.001	0.68 (0.57, 0.82) <0.001
Fever (≥37.8°C)	4 (4)	147 (3)	97 (4)	1.25 (0.39, 3.59) 0.565	1.60 (0.55, 4.65) 0.388	1.26 (0.96, 1.65) 0.095	1.21 (0.85, 1.73) 0.294
Vomiting	78 (80)	3774 (85)	2005 (86)	0.71 (0.42, 1.22) 0.233	0.74 (0.37, 1.46) 0.384	1.04 (0.90, 1.20) 0.599	1.34 (1.07, 1.66) 0.010
Diarrhea duration of >1 day	39 (40)	1618 (37)	1030 (44)	1.17 (0.76, 1.79) 0.528	1.55 (0.89, 2.71) 0.121	1.37 (1.23, 1.52) <0.001	1.52 (1.31, 1.78) <0.001
Stool frequency >10/24 hours	34 (35)	2024 (46)	1083 (46)	0.64 (0.41, 0.99) 0.046	0.69 (0.41, 1.17) 0.178	1.02 (0.93, 1.13) 0.655	0.94 (0.82, 1.08) 0.391
Watery stool	92 (95)	4153 (94)	2168 (93)	1.21 (0.47, 3.39) 0.849	1.33 (0.43, 4.17) 0.623	0.84 (0.69, 1.03) 0.095	1.07 (0.78, 1.46) 0.654
Abdominal pain	49 (51)	2635 (60)	1458 (62)	0.69 (0.46, 1.06) 0.091	0.67 (0.41, 1.10) 0.115	1.13 (1.02, 1.25) 0.024	1.12 (0.97, 1.28) 0.097
Dehydration (some or severe)	79 (81)	3355 (76)	1764 (76)	1.40 (0.82, 2.43) 0.246	3.12 (1.52, 6.41) 0.002	0.98 (0.87, 1.11) 0.780	0.97 (0.79, 1.19) 0.765
IV rehydration	38 (39)	2539 (57)	1327 (57)	0.48 (0.31, 0.73) <0.001	0.46 (0.25, 0.84) 0.011	0.98 (0.88, 1.08) 0.644	0.94 (0.78, 1.13) 0.502
Hospitalization >24 hrs.	16 (17)	861 (20)	599 (26)	0.82 (0.46, 1.44) 0.548	1.13 (0.58, 2.18) 0.728	1.43 (1.26, 1.61) <0.001	1.51 (1.28, 1.77) <0.001
Isolation of pathogens							
* V. cholerae*	23 (24)	1677 (38)	932 (40)	0.51 (0.31, 0.83) 0.006	0.59 (0.33, 1.09) 0.094	1.09 (0.98, 1.21) 0.117	1.03 (0.88, 1.20) 0.694
* Shigella*	7 (7)	290 (7)	212 (9)	1.11 (0.47, 2.50) 0.957	0.67 (0.23, 1.95) 0.464	1.42 (1.18, 1.72) <0.001	1.34 (1.03, 1.73) 0.030
Rotavirus	9 (9)	143 (3)	66 (3)	3.06 (1.41, 6.43) 0.004	2.96 (1.21, 7.24) 0.017	0.87 (0.64, 1.18) 0.397	0.792 (0.53, 1.19) 0.261
* Salmonella*	1 (1)	91 (2)	45 (2)	0.50 (0.03, 3.33) 0.730	0.55 (0.071, 4.17) 0.560	0.94 (0.64, 1.36) 0.784	0.78 (0.48, 1.27) 0.322
* Compylobactar*	7 (7)	295 (7)	154 (7)	1.09 (0.46, 2.46) 0.992	0.92 (0.33, 2.59) 0.880	0.99 (0.80, 1.21) 0.944	0.99 (0.77, 1.28) 0.942

N.B: Well-nourished considered as reference category.

Conversely, use of antimicrobials before coming to the hospital, longer duration of diarrhea (>1 day), vomiting, rehydration method used such as intravenous infusion of saline, and *V. cholerae*, *Shigella*, *Campylobactar* and rotavirus were found to be associated with overweight and obesity both in univariate as well as in multinomial logistic regression analysis with the exception of *V. cholerae* among those more than 19 years old compared to well-nourished and malnourished individuals (Table 3). Moreover, significant association was found with dehydration status in multinomial analysis which was not associated in univariate analysis (Table 3).

**Table 3:** Comparative and multinomial logistic regression analysis of characteristics and common etiology of diarrhea among well-nourished with overweight and obese, and malnourished individuals aged above 19 years.

Socio-demographic characteristics	Overweight and obese n = 1,330 (%)	Well-nourished n = 8,497 (%)	Malnourished n = 6,223 (%)	Overweight and obese vs. well-nourished		Malnourished vs. well-nourished	
				Unadjusted OR (95% CI) p value	Adjusted OR (95% CI) p value	Unadjusted OR (95% CI) p value	Adjusted OR (95% CI) p value
Antimicrobial therapy before attending the hospital	465 (35)	1804 (21)	872 (14)	1.99 (1.76, 2.26) <0.001	1.86 (1.58, 2.18) <0.001	0.62 (0.55, 0.66) <0.001	0.59 (0.54, 0.66) <0.001
Fever (≥37.8°C)	34 (3)	218 (3)	160 (3)	1.00 (0.68, 1.46) 0.941	0.85 (0.54, 1.33) 0.478	1.00 (0.81, 1.24) 0.975	1.08 (0.84, 1.36) 0.531
Vomiting	916 (69)	6455 (76)	4877 (78)	0.70 (0.62, 0.80) <0.001	0.85 (0.71, 1.03) 0.106	1.15 (1.06, 1.24) <0.001	1.08 (0.95, 1.21) 0.235
Diarrhea duration of >1 day	601 (45)	3069 (36)	2315 (37)	1.46 (1.30, 1.64) <0.001	1.24 (1.05, 1.47) 0.012	1.05 (0.98, 1.12) 0.184	0.17 (1.05, 1.30) 0.003
Stool frequency >10/24 hours	747 (56)	4603 (54)	3361 (54)	1.08 (0.96, 1.22) 0.184	1.09 (0.93, 1.27) 0.277	0.99 (0.93, 1.06) 0.857	0.98 (0.89, 1.07) 0.699
Watery stool	1229 (92)	7914 (93)	5788 (93)	0.90 (0.72, 1.12) 0.358	1.05 (0.79, 1.40) 0.730	0.98 (0.86, 1.12) 0.786	0.98 (0.82, 1.19) 0.857
Abdominal pain	814 (61)	5288 (62)	3999 (64)	0.96 (0.85, 1.08) 0.490	0.94 (0.80, 1.09) 0.391	1.09 (1.02, 1.17) 0.012	1.10 (1.01, 1.20) 0.037
Dehydration (some or severe)	999 (75)	6571 (77)	4936 (79)	0.88 (0.77, 1.01) 0.079	1.31 (1.07, 1.61) 0.008	1.12 (1.04, 1.22) 0.004	1.12 (0.97, 1.27) 0.111
IV rehydration	549 (41)	4452 (52)	3525 (57)	0.64 (0.57, 0.72) <0.001	0.57 (0.47, 0.69) <0.001	1.18 (1.11, 1.27) <0.001	1.11 (0.99, 1.24) 0.066
Hospitalization >24 hrs.	247 (19)	1685 (20)	1583 (25)	0.92 (0.79, 1.07) 0.299	1.08 (0.88, 1.35) 0.484	1.38 (1.27, 1.49) <0.001	1.32 (1.19, 1.47) <0.001
Isolation of pathogens							
* V. cholerae*	271 (20)	2008 (24)	1580 (25)	0.83 (0.72, 0.96) 0.010	1.05 (0.86, 1.29) 0.620	1.10 (1.02, 1.19) 0.015	1.07 (0.96, 1.19) 0.244
* Shigella*	49 (4)	483 (6)	470 (8)	0.63 (0.46, 0.86) 0.003	0.46 (0.31, 0.68) <0.001	1.36 (1.19, 1.55) <0.001	1.39 (1.16, 1.67) <0.001
Rotavirus	84 (6)	339 (4)	175 (3)	1.62 (1.26, 2.09) <0.001	1.50 (1.09, 2.05) 0.011	0.70 (0.58, 0.84) <0.001	0.93 (0.74, 1.18) 0.552
* Salmonella*	30 (2)	176 (2)	118 (2)	1.09 (0.72, 1.64) 0.738	1.17 (0.74, 1.86) 0.495	0.91 (0.72, 1.16) 0.490	0.88 (0.65, 1.22) 0.460
* Compylobactar*	37 (3)	411 (5)	322 (5)	0.56 (0.39, 0.80) 0.001	0.58 (0.38, 0.88) 0.011	1.07 (0.92, 1.25) 0.373	1.13 (0.94, 1.36) 0.207

N.B: Well-nourished considered as reference category.

**Figure 3:**
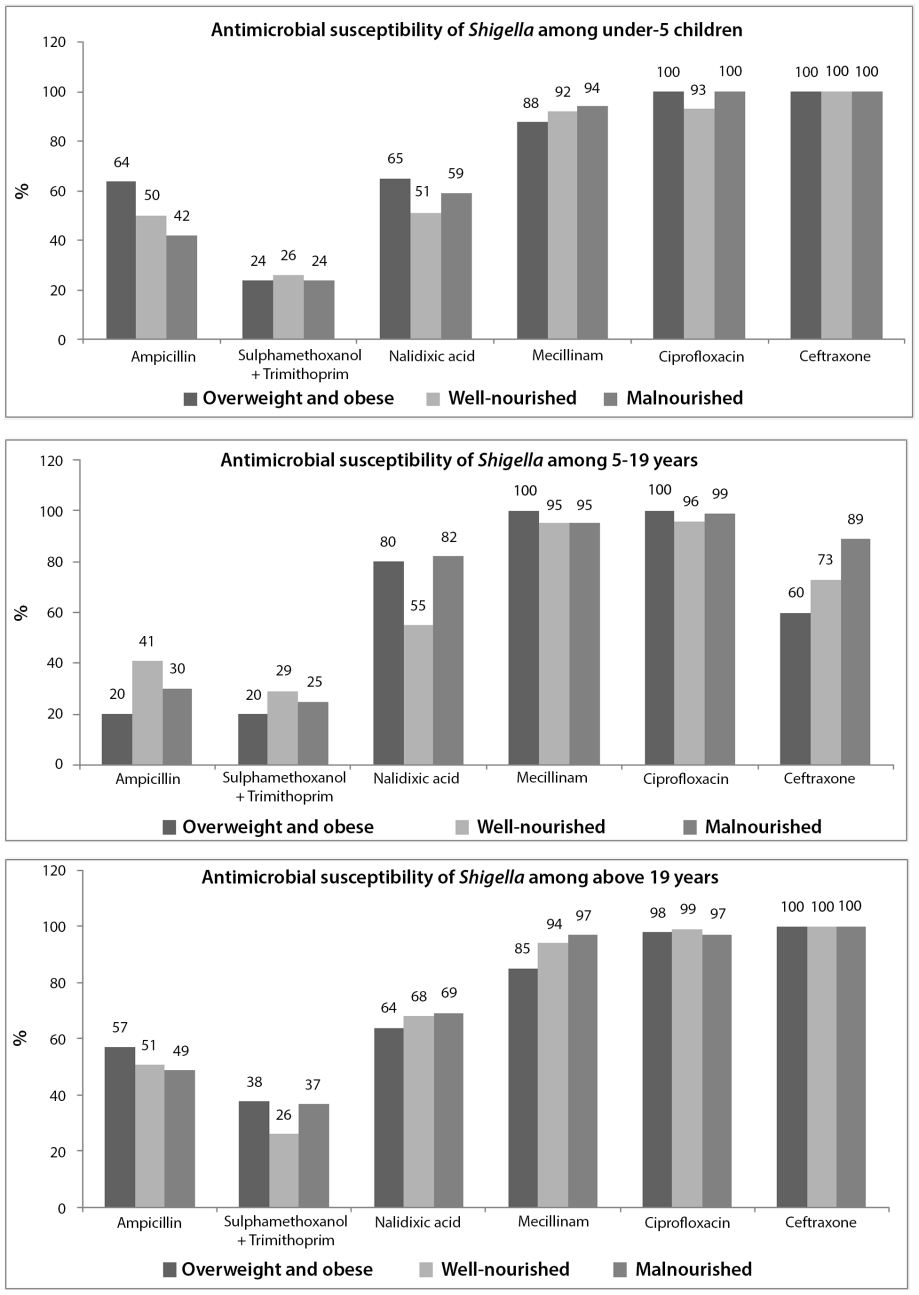
Distribution of antimicrobial susceptibility of *Shigella* among three age stratum.

In terms of antimicrobial susceptibility, 93 to 100% of the *Shigella* isolates were susceptible to ciprofloxacin irrespective of age and nutritional status. Although 100% susceptibility was observed for ceftriaxone for under-5 children and those 19 years and above, among 5–19 years it was 60%. The susceptibility for mecillinum ranged from 85% to 100% among overweight and obese group (Figure 3); however, for well-nourished and malnourished groups it ranged between 92% to 97%. For ampicillin, trimethoprim-sulfathmethoxazole (TMP-SMX), and nalidixic acid susceptibility ranged from 20%–64%, 20%–38% and 64%–80% among overweight and obese individuals correspondingly and it was irrespective of age (Figure 3).

**Figure 4:**
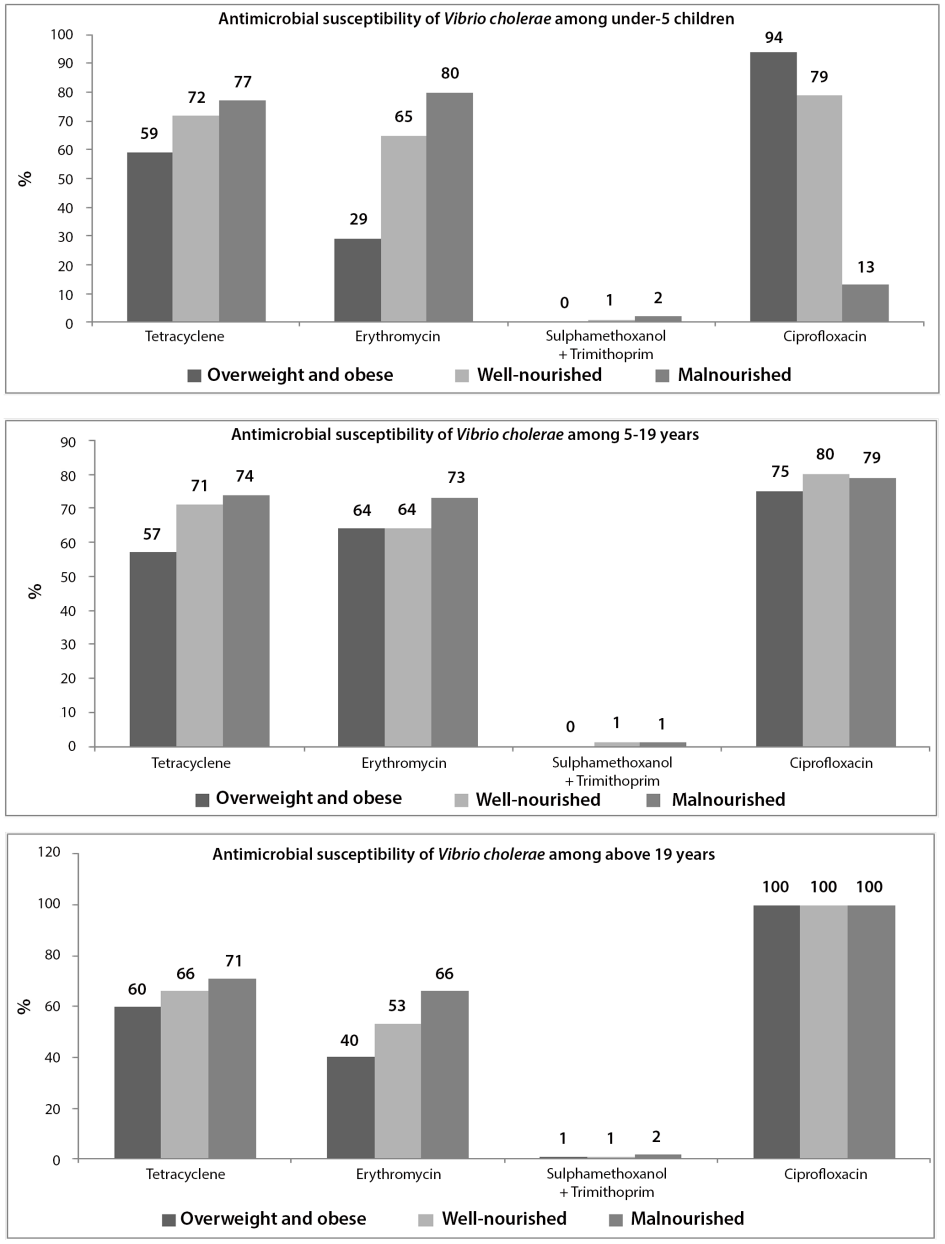
Distribution of antimicrobial susceptibility of *Vibrio cholerae* among three age stratum.

For *Vibrio cholerae*, 100% susceptibility was found only for ciprofloxacin among those aged above 19 years; however, for under-5 children, and 5–19 years old, it was 94% and 75% respectively (Figure 4). For well-nourished and malnourished, it ranged between 79% and 100%; and 13% to 100% respectively with variations in age. Susceptibility to erythromycin and tetracycline among overweight and obese individuals ranged between 29%–64% and 57%–60% among those from three age stratums (Figure 4).

## Discussion

Diarrhea is a major public health problem in Bangladesh. Differences in the prevalence, etiology, clinical presentation, complications, and outcome between well-nourished and malnourished individuals have been well described in medical literatures [31]. However, there was no information describing these differences in those who are overweight and obese. Given the increasing rates of obesity in low-income settings such as Bangladesh, addressing this knowledge gap is an important contribution to the study of diarrheal disease surveillance.

Earlier studies indicated a higher rate of isolation of rotavirus from well nourished children than their malnourished counterparts [32]. This study found that the rate of isolation of this pathogen was the highest among overweight and obese irrespective of age. On the other hand, the overall proportion of rotavirus increased from 20% to 44% among all under-5 children from 1993 to 2011. The surge of rotavirus was even higher among overweight and obese children than other groups at each point of observation except 1998. The observation of higher rate of rotavirus isolation among overweight and obese under-5 children is difficult to explain. Increased flooding over time in all over Bangladesh including Dhaka may have had an overall impact on increased number of patients presenting with this pathogen, as well as changes in the distribution of etiologic agents like excess number of cholera cases which might have outnumbered the presentation of rotavirus cases in excess. [33].

Higher rates of rotavirus among well-nourished individuals may be related to healthy epithelial cells that are required for attachment of this pathogen as well as for the development of rotavirus diarrhea [34]. It is likely that obese and overweight children are not suffering from other diseases of the digestive tract that may present in underweight children. Another explanation could be differences in the hygienic practices of families of children who are overweight and obese. In Bangladesh, it may be that overweight and obese children are from families with a better socio-economic status, and this population may have different eating behaviors, which may be in turn responsible for differed likelihood of developing food-borne diseases. A propensity for consuming food away from home may lead to increased consumption of food prepared with poor food hygiene practices. On the other hand, overweight and obese individuals were less likely to be infected with *Shigella* compared to malnourished individuals. Malnourished children have higher risk for shigellosis [32], and the present observation was also identical with the previous studies [35].

Use of antimicrobials prior to hospitalization was significantly higher among obese and overweight individuals in both univariate as well as multivariate analyses. Better socio-economic conditions, more frequent prior consultations with physicians and higher purchasing capacity as well as client demand for drugs, and self-purchase of antimicrobials by them could explain this observation.

In the present study, the antimicrobial susceptibility to commonly used antimicrobials for treatment of diarrheal disease illnesses was different among different age groups and in different nutritional status groups. Presumably the uses of antimicrobials are dependent on pathogen isolation, the isolation is different in different age groups; and the selective pressure also varies showing a variable susceptibility pattern. Malnutrition enhances translocation of pathogens in the intestine and also impacts on immune system that might lead to diverse susceptibility to antimicrobials, which may have contributed to this. Use of antimicrobials is also associated with minimum inhibitory concentration (MIC) of antimicrobials against certain pathogens [36].

Inappropriate or early use of antimicrobials by overweight and obese individuals may have delayed hospital attendance among the older age group (above 19 years), and once again may have added to the burden for case management of severe cases presenting late with an associated increase in treatment problems or complications. This might be same for purchasing and using oral rehydration salts (ORS). However, such findings were similar among under-5 years and 5–19 years age groups, suggesting universal use of ORS for initial management of loss of fluid and electrolytes due to diarrhea. The proportion of dehydrating diarrhea among overweight and obese individuals in all age groups was low compared to well-nourished and malnourished. Higher rates of antimicrobials prior to receiving treatment and higher use of ORS may help to explain the lower rate of intravenous saline use for correction of dehydration among overweight and obese individuals. Additionally, diagnosis of dehydrating diarrhea may be lower as it is sometimes difficult to assess the dehydration status among this population following standard guidelines due to presence of excess subcutaneous fat. However, subcutaneous fat rarely affects appropriate assessment of dehydration status while there are well defined guidelines to assess dehydration in a tertiary level hospital.

Rapidly increasing prevalence of childhood obesity (5–19 yr) in developing countries like 41.8% in Mexico, 22.1% in Brazil, 22.0% in India, and 19.3% in Argentina might be a public health concern [37], so is the case for Bangladesh [37]. In addition to long sufferings, diarrhea is often making this population at risk for poor health care delivery challenge, especially proper assessment of dehydration status and its subsequent management. On the other hand, reporting of increased number of overweight and obese patients in the triage area may alert clinicians and policy makers to take necessary measures to control the burden of this problem [14].

### Limitations

Our study was conducted among people attending a hospital, who may not be representative of the general population. However, the hospital provides cost-free treatment and it is accessible to all people, irrespective of their socioeconomic or other status; and the systematic, large sample and unbiased sampling of the surveillance system are the strengths of the study. Genetic interactions might play an important role in making this population vulnerable to altered or compromised metabolic functions which may influence optimal immune responses among this population. Obesity is known to influence non-specific and specific immune functions mediated by humoral and cell mediated mechanisms. However, the present study was not designed to determine the immune responses as well as socio-demographic and behavioral characteristics among these urban and peri-urban overweight and obese population. Lack of information examining other associated co-morbidities such as pneumonia may be another limitation.

### Conclusions

Increased prevalence of overweight and obesity are now impacting global health, and Bangladesh is not beyond this health threat. Diarrhea may contribute to the morbidity burden among this population, and proper assessment of dehydration status and its subsequent management may differ in this population. The obvious increase in the number of overweight and obese patients in the triage area found in this study should alert clinicians and policy makers to take necessary measures to control the burden of diarrheal disease among this population.

In the present study, obese and overweight individuals had a varied susceptibility to various pathogens which cause diarrheal diseases. The causes of these differences are unclear, however with changing trends in nutritional status in Bangladesh, it is important for those addressing diarrheal disease to consider these influential factors. Moreover, further studies as well as continued disease surveillance should be prioritized to minimize the knowledge gap in host characteristics that predict susceptibility of obese and overweight population to diarrheal illnesses.
